# The New Tics study: A Novel Approach to Pathophysiology and Cause of Tic Disorders

**DOI:** 10.20900/jpbs.20200012

**Published:** 2020-05-27

**Authors:** Kevin J. Black, Soyoung Kim, Bradley L. Schlaggar, Deanna J. Greene

**Affiliations:** 1 Departments of Psychiatry, Neurology, Radiology and Neuroscience, Washington University in St. Louis School of Medicine, St. Louis, MO 63110, USA; 2 Departments of Psychiatry and Radiology, Washington University in St. Louis School of Medicine, St. Louis, MO 63110, USA; 3 Kennedy Krieger Institute, Baltimore, MD 21205; and Departments of Neurology and Pediatrics, Johns Hopkins University School of Medicine, Baltimore, MD 21287, USA

**Keywords:** Provisional Tic Disorder, tic disorders, Tourette syndrome, prognosis, anxiety, tic suppression, inhibition (psychology), hippocampus, MRI

## Abstract

We report on the ongoing project “The New Tics Study: A Novel Approach to Pathophysiology and Cause of Tic Disorders,” describing the work completed to date, ongoing studies and long-term goals. The overall goals of this research are to study the pathophysiology of Provisional Tic Disorder, and to study tic remission (or improvement) in a prospective fashion. Preliminary data collection for the project began almost 10 years ago. The current study is nearing completion of its third year, and has already reported several novel and important results. First, surprisingly, at least 90% of children who had experienced tics for only a mean of 3 months still had tics at the 12-month anniversary of their first tic, though in some cases tics were seen only with remote video observation of the child sitting alone. Thus almost all of them now had a DSM-5 diagnosis of Tourette’s Disorder or Persistent (Chronic) Tic Disorder. Baseline clinical features that predicted 12-month outcome included tic severity, subsyndromal autism spectrum symptoms, an anxiety disorder, and a history of 3 or more phonic tics. Second, we found that poorer tic suppression ability when immediately rewarded for suppression predicted greater tic severity at follow-up. Third, striatal volumes did not predict outcome as hypothesized, but a larger hippocampus at baseline predicted worse severity at follow-up. Enrollment and data collection continue, including functional connectivity MRI (fcMRI) imaging, and additional analyses are planned once the full sample is enrolled.

## INTRODUCTION

### Background

Tics are brief movements or noises, repeated many times a day that may look intentional but serve no useful purpose [1]. Common tics include forceful blinking, raising the eyebrows, turning the head, shrugging, sniffing, or grunting. Tics in a few patients are attributable to another disease, such as stroke or Huntington disease, but usually no such secondary cause is found [2]. Tics almost always begin in childhood, most often ages 3 to 10 years, and on average, tics are most severe around ages 9 to 11 [3]. Chronic tic disorders are defined by the persistence of tics for at least 12 months (meaning tics collectively, because individual tics come and go over time). If both movements and vocalizations have been present, childhood-onset idiopathic chronic tics are diagnosed as Tourette’s Disorder (Tourette syndrome, TS), whereas if all tics are silent the diagnosis is Persistent (Chronic) Motor Tic Disorder (CTD) [4,5]. The terms and definitions have changed somewhat over time, and for children who have had tics for less than a year, the nosology has been confusing. For instance, DSM-IV, DSM-IV-TR, and DSM-5 define quite different, though overlapping, groups of children with tics for less than a year (see Appendix 1 in ref. [6]). The current nosology defines Provisional Tic Disorder (PTD) by the presence of tics not better explained by another brain or systemic illness, with no tic prior to 1 year ago.

Despite increasingly intensive efforts, our understanding of the etiology and pathophysiology of TS/CTD remains fragmentary [7–9]. Furthermore, studies that find an association of TS with a particular biological marker usually are unable to determine whether the association is causal, and if so whether the marker is a consequence or cause of the disease. Treatment is entirely symptomatic, and despite ongoing discovery efforts, many patients remain frustrated with the limited efficacy of available treatments [10]. In short, in reviewing this state of affairs, we concluded that a new approach to the study of tic disorders might be needed. The new approach was suggested by epidemiological data.

A number of careful, independent epidemiologic studies have shown that chronic tic disorders affect at least 2–3% of children, including TS in about 0.5–1% of all children 5–14 years old [11]. These rates may be underestimates: in one study, after parent, teacher and child had reported no tics in 8 children, and an expert visiting the classroom had observed no tics, tics were nevertheless observed during an office visit in 3 of the 8 [12]. In any case, it clearly appears much more common for children to have tics for less than the 12 months required for a diagnosis of TS/CTD. Several studies that used direct clinical ascertainment reported tic prevalence as ~20% [13,14], a rate also found in groups of younger children only [15]. One of the most careful studies to date used a defined population, stratified sampling, assessment procedures with tested reliability, and personal examination by a neurologist of all probable cases and of a number of screen-negatives [16]. This study found a DSM-IV-TR tic disorder in 17% of mainstream school children ages 6–17. In another study, during 8 visits over a single school year, trained raters directly observed a motor tic in 24% of all children in an elementary school, including 49% of all first-graders [17]. Our recent review concluded that most children probably have a tic at some point in time [6]. So what happens to most of these children with tics, if tics last for a year in only ~2–5% of children? Clinical lore suggests that most children who just started ticcing will remit within a few months [5,18–25]. However, lore is not data.

Remarkably, almost no research has been done on this “pre-Tourette” population, setting up the scientific premise of the current research study. Prospective outcome data are sparse and contradictory [6]. Shapiro et al reported outcomes based on selected clinical features [24] (p. 371–374 and Table 10 in Chapter 12). Bruun and Budman reported telephone follow-up on 58 children who had presented with tics lasting less than a year [26]. Carter and colleagues [27] performed a 4-year high-risk follow-up study in first-degree relatives of TS probands. Spencer et al prospectively monitored the appearance and disappearance of tic disorders over the course of 4 years in boys with or without ADHD who did not have Tourette syndrome at study entry [28]. Peterson et al. [15] followed for up to 15 years a large sample of children with chronic or recent-onset tics, diagnosed initially by parental report only; the follow-ups used direct examination for diagnosis. A few other studies report limited prospective data [29–31]. However, all these studies report only clinical information at baseline. Several superb studies did follow patients *after* tics were chronic [32–40]; although these follow-up studies of chronic tics do not address why some tics fade before TS can be diagnosed, they help identify reasonable hypotheses [6].

Research has understandably focused on those with chronic tics. But ironically, there are important questions about TS that cannot be addressed in people already diagnosed with TS. For instance, are there abnormalities of brain function when tics first appear? Why do tics usually disappear? What is different about children whose tics persist? And perhaps of greatest interest, could early intervention in those at highest risk prevent tics from becoming chronic? The present study targets just these questions.

Another way of framing the problem is to realize that two steps occur in every child who develops TS/CTD: first tics appear; then they fail to remit. Given the 10-fold difference in accepted prevalence between PTD and TS/CTD, the second step may be more important. Importantly, this second step can be observed prospectively, minimizing the biases inherent in retrospective study designs. Equally important, studying persistence vs. remission may identify causative factors missed by studies of established TS, in which onset and persistence are confounded and often remote. Studying this earliest phase of tic disorders may revolutionize etiology, prevention or treatment.

Studying pathophysiology of recent-onset tics has been considered laudable but impracticable, as recent-onset tics may not be identified as such nor bring the child to medical attention. For instance, in one study the estimated delay between tic onset and first clinical contact was 10 years [41]. However, by intensive recruitment efforts, we have been able to recruit at an adequate pace children who had tics for less than 6 months (more recently allowing up to 9 months; hereinafter “New Tics”). We now have 12-month outcome data on 63 children with New Tics. We have discovered fascinating clinical results, and are beginning to identify biological predictors of outcome.

### Impact

Chronic tic disorders have substantial public health significance [23], and the proposed research may have substantial benefits for TS/CTD. The clearest benefits are that it may identify entirely new directions for further etiologic and pathophysiological research, and it can clarify the causality of associations we find.

Take new directions first. In addition to the specific hypotheses we are testing, we will perform data-driven analyses to identify potential new findings. Newly identified brain regions can later be studied with electrophysiological methods or in the limited available number of TS autopsy specimens. The information this study can provide on causality is also very important. Take the example of brain regions whose volume is smaller in adults with TS compared to control adults. This finding may indicate areas whose development was atypical, causing tics to develop. Alternatively, they could be compensatory changes to help suppress tics [42,43]. Or, both volumetric changes and clinical symptoms could reflect some other root cause. Cross-sectional studies generally cannot distinguish these three possibilities. By contrast, the proposed prospective study, starting before tics become chronic, can isolate the disappearance of tics. For example, brain regions in which abnormal volume at baseline predicts improvement of tics have a much clearer causative link with tic remission [36]. As another example, abnormalities in TS/CTD that are also present in the New Tics group are unlikely to reflect chronicity or adaptation.

The New Tics population is also important in its own right. Children with recent-onset tics are seen commonly in pediatrics or child neurology practices. “Most parents want predictions of the future for their child,” wrote one clinician, adding, “This, however, is one of the more challenging aspects of caring for the child with [tics]” [18]. Clinical lore is that new-onset tic disorders usually remit, but published follow-up studies tell a different story: the mean remission rate is only 32% (Table 1 in ref. [6]). In the largest such study, children whose tics disappeared within the first year were followed up after 2–14 years, and only 17% had remained tic-free throughout [26]. Regardless of the true remission rate, some New Tics children do go on to have years of clinically problematic tics. Since at present there is almost no information to determine which of them will need long-term treatment, the proposed study will be important for the clinical outcome data alone.

More importantly, this population offers the tantalizing possibility of *prevention* of TS (or, if one prefers, secondary prevention of tic disorders). Now that large randomized, controlled trials have demonstrated the efficacy of CBIT, a specific behavior therapy for tics with no known side effects [44], it becomes entirely plausible to envision providing CBIT to those children with New Tics who are at highest risk of TS/CTD. Conceivably such early treatment could prevent progression to TS. The proposed work takes preliminary but necessary steps toward that possibility.

We include the neurobiological measures to help us begin to understand the mechanisms of tic disappearance. If we understood those mechanisms better—if we knew *why* tics improve in many of these children—we could hope to design rational, mechanism-based treatments for patients who do have persistent tics. Biomarkers that predict remission of New Tics may conceivably speed medication discovery. They may even identify *targets* of treatment. For instance, the association of ADHD at baseline with later tic persistence [15] may indicate aggressive treatment of ADHD in new tic patients (or may not; as with other links we may uncover to tic outcomes, this association may not indicate causation and will need to be tested).

### Hypotheses and Aims

Here we discuss the hypotheses and specific aims for the project currently funded by the National Institutes of Health (NIH) as “The New Tics Study: A Novel Approach to Pathophysiology and Cause of Tic Disorders” (grant R01MH104030). Briefly, we hypothesized that specific features would differentiate the New Tics participants from tic-free controls, and predict worse outcome at the one-year anniversary of tic onset (the accepted minimum duration for diagnosis of TS/CTD).

Given the paucity of knowledge in this area and the substantial effort required to recruit these children, we are collecting a rich set of data. However, to ensure rigor and reproducibility we focused our analyses on a limited number of hypotheses that the available evidence best supports.

The available literature on New Tics suggests a few hypotheses for our proposed large, prospective study [6]. In a previous prospective study, tic disorders were much more common in boys with ADHD (37% by study end) than in boys without ADHD (7%) [28]. Similarly, ADHD at baseline was associated with later tic persistence [15]. In a cross-sectional prevalence study, children with tics for less than a year had lower severity, later age of onset, and were less likely to have vocal tics than did children with TS or CTD [45]. Our preliminary data suggest that children with New Tics who can suppress tics better to verbal request may have better outcome at the 12-month anniversary of tic onset [46].

Although only indirectly relevant, many more studies have examined children *after* tics have become chronic, and these studies suggest some relevant hypotheses about New Tics.

Children who were less able to suppress tics, with or without immediate reward, tended to have more severe symptoms [47]. Omission errors on the Connors’ Continuous Performance Test (CPTII) were negatively correlated (*r* = −0.63) with suppression ability [48]. About half of TS adolescents exceeded the clinical cut-off point for hyperactivity and anxiety on the Child Behavioral Checklist (CBCL) subscales even though only 22.5% met DSM-IV criteria for ADHD and 7.5% met criteria for anxiety disorders [49]. Relatively impaired manual dexterity (Purdue Pegboard test) was associated with outcome an average of 8 years after diagnosis of TS [50]. Subjects with TS performed significantly worse on a “weather prediction” probabilistic classification test, a form of habit learning [51].

Kurlan et al. noted that “there seems to be a strong relationship between the experience of premonitory sensations and the ability to suppress tics” [52]. That relationship was recently confirmed empirically [53]. In addition, premonitory urges are generally reported later in the course of TS than are simple motor tics, and they are associated with thinner insula and sensorimotor cortex [54]. More relevant, in TS, tics preceded by a premonitory urge tended to remain worse after psychotherapy [55]. One can reasonably argue about the direction of the association, but premonitory urges are likely to be important in predicting tic outcome.

When our grant proposal was submitted, reduced caudate volume was arguably the most consistent structural neuroimaging finding in TS [9]. The likely etiological relevance of this finding was shown by a study of 43 children with established TS, in which a smaller caudate nucleus in childhood predicted more severe tics and other symptoms an average of 7.5 years later [36]. A number of other structural changes have been implicated in TS, including smaller ventral prefrontal cortex (vPFC), thinning of sensorimotor cortex, and increased gray matter volume in thalamus and midbrain [56,57].

A handful of studies used [^99*m*^Tc]HMPAO SPECT to measure blood flow in TS, as reviewed elsewhere [9]. Two of the largest included 38 children with TS and 18 controls [58] and 50 children with TS and 20 controls [59]. Decreased left caudate blood flow was found in most studies, including both of these. Decreased flow in prefrontal regions was also common. Blood flow can also be measured with MRI using arterial spin labeling (ASL). The most important potential advantage of ASL over BOLD (blood oxygen level-dependent fMRI) is its signal stability over time: comparisons of brain activity between two conditions (such as at screening versus at follow-up) can be performed most directly using perfusion imaging. We are not aware of published studies applying ASL imaging to TS, but we have substantial prior experience with ASL in other patient populations [60–63].

We previously published data from a resting state functional connectivity fMRI (rs-fcMRI) experiment in 33 children with TS (DSM-IV-TR), age 10–16, and 42 controls [64,65], and compared results to over 200 healthy subjects from age 7 through adulthood [66]. However, more recently we and others have documented substantial artifactual effects of even small, repeated head movements on fcMRI [67], and our site has been a leader in developing analysis methods that avoid such artifacts to the limits of detection [68–70]. We recently reported results of an rs-fcMRI study of TS in which we applied the best available methods to minimize movement-related artifact [71]. We applied a multivariate machine learning technique—support vector machine (SVM) classification—to test whether patterns in brain network activity, measured with rs-fcMRI, differed between 42 children with TS (8–15 years) and 42 unaffected controls matched for age, IQ, and in-scanner head movement. Univariate tests identified no significant group differences, but SVM could distinguish TS from control brains with ~70% accuracy (*p* < 0.001). These results suggest that multivariate methods can better capture the complexity of some brain disorders. The pattern of brain activity that distinguished the TS and control groups was complex, but rs-fcMRI within and between somatomotor and higher-level control networks (fronto-parietal, cingulo-opercular, salience, dorsal attention, and ventral attention networks) produced classification reliability closest to that of the complete data set. Other investigators have demonstrated improved SVM classification accuracy by combining different data types [72,73], so we expect improved accuracy by combining rs-fcMRI, structural MRI, and behavioral/clinical data in the current study.

Bohlhalter et al. identified brain regions in which BOLD signal began to increase during the 2 seconds *prior* to a tic [74]. These regions included supplementary motor cortex (SMA), known to be involved in movement generation, as well as anterior cingulate (ACC) and insular regions that have been implicated in processing unpleasant sensations. Hampson et al found that the SMA activation begins earlier and persists longer than SMA activation in controls voluntarily performing movements similar to patients’ tics [75].

Building on previous work in adults [76], we described developmental changes in functional connectivity between basal ganglia voxels and functionally defined rs-fcMRI cortical systems [77]. Cortical-basal ganglia connections differed most significantly between children and adults in the functional connectivity of the posterior putamen and pallidum with the somatomotor “face” network. This correlation decreased significantly from ages 7 to 13 years, and remained low throughout adulthood. We hypothesize that the higher correlation seen in younger children may also be a marker for tic diagnosis or severity.

The deficient emotional self-regulation profile from the Child Behavior Checklist (CBCL-DESR) consists of scores on the Anxiety/Depression, Aggression and Attention subscales of the CBCL [78,79]. This scale was intended to be sensitive to dysregulation in multiple domains, including affect (anxiety/mood), behavior (disruptive), and cognition (attention) and has been thought to represent a global deficit in self-regulation related to processes of effortful control as defined by Eisenberg and Spinrad [80]. Although this subscale has not been reported in TS, it is postulated to associate with deficient central inhibitory processes, which may predict greater difficulty suppressing tics.

#### Summary of hypotheses

Of the numerous reasonable hypotheses one could make about a New Tics sample [6], we focus on five primary hypotheses to limit Type I error. We propose that the following features are more likely to be present in the New Tics group than in the tic-free control group (**Aim 1a**, below), will be associated with worsening of tics (or less improvement in tics) at 12 months after tic onset (**Aim 2a**), and if present at baseline will predict worse 12-month outcome (**Aim 2b**).

1. Lesser ability to suppress tics to verbal request (Tic Suppression Paradigm)
2. More omission errors during sustained attention (Continuous Performance Test)
3. Smaller caudate nucleus after correction for total brain volume
4. Greater severity of tics at screening (YGTSS total tic score, rated after observation during the Tic Suppression Paradigm)
5. More premonitory urges (higher score on the PUTS scale)

In addition to this disciplined hypothesis-testing approach, given that so little work has been done in this area, we will also examine the full set of data using machine learning methods. Specifically, we will perform SVMs (for group comparisons) and SVRs (for correlations in **Aim 2**). Using the full data set (clinical, neuropsychological, and structural and functional MRI), we will perform SVMs for group comparisons and SVRs for correlations with improvement in YGTSS total tic score (TTS), as an indication of clinically relevant symptom change from the screening visit to the 12-month visit. We will then interrogate the SVM and SVR results to identify features that drive the group differences or correlations, to identify novel or multivariate findings relevant to pathophysiology or prognosis. This data-driven approach to hypothesis generation motivates our collecting the other planned clinical and imaging data.

**Aim 1.** Study pathophysiology of recent-onset tics.

**Aim 1a.** Identify clinical, neuropsychological, and brain imaging features that differentiate children with recent tic onset (“New Tics” group) from tic-free controls. We will test *a priori* hypotheses including tic suppression, inattentiveness, caudate nucleus volume, tic severity, and premonitory urges (see “Summary of hypotheses”). Secondary analyses will apply support vector machine (SVM) learning to a rich set of data to discover novel, multivariate differences in the New Tics group [71,81]. These data will also include tic phenomenology, psychiatric diagnosis, habit learning, motor dexterity, structural MRI, perfusion MRI, and rs-fcMRI.

**Aim 1b.** Compare New Tics subjects to a group of children who are matched for age but have already had tics for ≥1 year (“Existing TS/CTD”). Since both groups have tics, this comparison will highlight abnormalities that cannot be explained by the mere current presence of tics, including markers of chronicity or adaptation.

**Aim 2.** Prospective study of tic remission.

We will reevaluate New Tics subjects at the 1-year anniversary of tic onset. The primary dependent variable was chosen to be the change in tic symptom severity (ΔTTS = change in YGTSS Total Tic Score from baseline to follow-up). We focus on outcome as a continuous variable because no reliable estimate exists for how many New Tics subjects will remit versus go on to diagnosis with TS/CTD. Remission rate also depends on definition and on the thoroughness of the follow-up evaluation [6].

**Aim 2a.** Study the physiology of tic remission by identifying changes in clinical, neuropsychological, and brain imaging variables that correlate with changes in clinical tic severity (ΔTTS). This Aim benefits from prospective observation and within-subject comparisons. The primary analysis will focus on any markers identified in Aim 1. A secondary analysis will apply machine learning methods for a data-driven approach (support vector regression, SVR).

**Aim 2b.** Identify predictors of improvement or worsening, i.e., clinical, neuropsychological, and brain imaging features at study entry that correlate significantly with ΔTTS. The 2 primary analyses will relate clinical outcome (ΔTTS) to tic suppression ability and caudate volume at study entry. Secondary analyses will examine other predictors using an SVR machine learning approach.

## MATERIALS AND METHODS

### Overview

The original grant proposals, and the current study protocol with additional methodological details, are archived on the Open Science Framework at https://osf.io/y5vxj/.

The work described herein was approved by the Washington University Human Research Protection Office (IRB), protocol numbers 201109157 and 201707059. Each child assented and a parent (guardian) gave informed consent. Data shared from other projects were shared after appropriate human subjects review and consent.

New Tics subjects have clinical, neuropsychological and MRI assessments within 6 months of tic onset and again at the 12-month anniversary of their first tic. Tic-free control children, matched to the New Tics subjects on age, sex, handedness, and if possible ADHD, will have clinical and MRI assessments at screening and a clinical follow-up visit without MRI. Children with Existing TS/CTD will likewise be matched to the New Tics subjects and have MRI at baseline and clinical follow-up without MRI.

**Figure 1.**
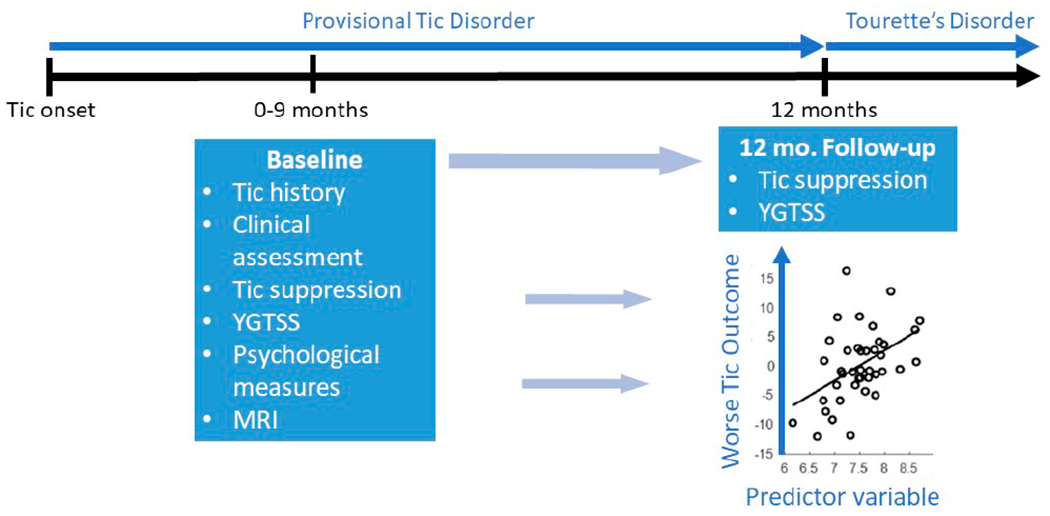
Overall study design. Children are examined thoroughly at a baseline visit no more than 9 months after their first tic (median 3.5 months). The follow-up visit occurs as close as possible to the one-year anniversary of their first tic, when DSM-5 Tourette’s Disorder can first be diagnosed.

Figure 1 shows the general outline of initial study participation for children in the New Tics group. Briefly, children are brought in as soon as possible after their first tic. Effort is expended to identify as closely as possible the date of that first tic, as described in one of our early publications [82]. Thorough clinical assessment is accompanied by selected psychological tests and multimodal MRI (details are provided below). These results can be compared to those of tic-free control or existing TS/CTD participants to identify differences and begin to sort out their timing. The children then return at the 12-month anniversary of their first tic, when TS/CTD can first be diagnosed. Their clinical status at that visit, especially the total tic score from the YGTSS, allows us to determine which features present at the baseline visit best predict outcome at this follow-up visit a median of 3.5 months later.

Several important project features are not shown in Figure 1. First is that New Tics participants are followed up again, in person, at 1, 2 and 3 years after the 12-month follow-up visit. Second, the New Tics group has a second MRI session at 12 months, identical to their first session. Third, tic-free controls are not shown. Their participation is identical to that of the New Tics group, except that in-person visits end after the initial follow-up visit. Their “12-month” visit is timed to be the same duration after the baseline visit as that of the New Tics participant to whom they are best matched. Its primary purpose is to find out how many of them, if any, have developed tics during the follow-up period. Fourth, Existing TS/CTD participants are not shown in Figure 1; their participation is similar to that of the control group.

### Subjects

The primary study sample consists of 110 children with New Tics, including those previously studied and 70 newly enrolled subjects. We examine each of them within 9 months of tic onset using carefully selected clinical, neuropsychological, and MRI methods. They return at the 1-year anniversary of tic onset for clinical and MRI evaluation, and yearly thereafter for clinical follow-up only. For data analysis, the subjects studied in the pilot study will be added to those studied under this project.

To reduce unwanted variance in the sample, we limit our subjects to ages 5–10 at enrollment. This corresponds to the age of highest risk for onset of TS [3,71,83,84]. Chronic tic disorder and transient tic disorder also tend to start within this window (mean age of onset 7.4–8.5 years) [45]. The neuropsychological tests we are using are not normed for children under age 5, and very few 3- or 4-year olds could complete the awake MRI session. The upper age limit is arbitrary, but on a practical level, although our pilot study accepted children age 5–17, almost all subjects were age 6–9 at screening.

There are two comparison groups. The first consists of 70 tic-free control children who additionally have no first-degree relative with tics by parental history. We match these subjects 1:1 to New Tics subjects with good 12-month MRI data, based on age, sex and handedness. We also enrich this group for ADHD such that the proportion of tic-free children with ADHD will match that of the New Tics group. Most matches will be drawn from existing data from our labs; we have structural and rs-fcMRI studies from over 100 healthy controls [56,64–66,71]. Assuming a 74% rate of good MR scans in this group (estimated from our preliminary data) means scanning 54 new tic-free control children (41 with ADHD, 13 without) to yield 40 with good MRI data. Also, the added 54 control subjects will have identical diagnostic information as we will have on the New Tics group. These control subjects will also return for a “12-month” follow-up visit. Since they have no tic onset date to calculate the 12 months from, their follow-up visit will occur at the same time after screening as did the follow-up visit in their matched New Tics subject. The primary purpose of this clinical follow-up visit is to check whether or not they develop tics in the intervening months since screening. If needed, we can also request data from the TAA Neuroimaging Consortium, which has rs-fcMRI data from 88 TS subjects and 70 controls in addition to the data from our site, or from the Human Connectome Project, for which Dr. Schlaggar is a co-investigator.

The last group consists of 70 children who at the time of screening already have TS/CTD (“Existing TS/CTD group”). We will follow the same strategy as for the tic-free controls, leveraging existing data from our labs, including >100 children with structural and rs-fcMRI data. All of these subjects were personally examined by authors KJB or BLS, and additionally completed standard symptom rating scales. We add 25 new Existing TS/CTD subjects with good MRI data, requiring us to scan about 34. They also return for a clinical follow-up visit at the same interval after screening as their matched New Tics subject.

### Inclusion and Exclusion Criteria

Inclusion criteria for all subjects are: Age 5–10 at screening. New Tics group: tics now, but developed them only in the past 9 months. Exclusion criteria: secondary tics, another neurological disorder (not counting migraine), structural brain disease, severe systemic illness, lack of proficiency in the English language, and psychiatric illness including mental retardation, autism, substance dependence, current substance abuse, primary psychotic illness, bipolar disorder and current major depression. Psychoactive medications are allowed if their dose has not changed in the past month.

Inclusion criteria for Existing TS/CTD control group are: children who meet DSM-5 criteria for TS or CTD at enrollment, matched to children from the New Tics group on age (within 1 year), sex, handedness, and ADHD status. (Matching is not required if the children were thought to be in the New Tics group before the face-to-face screening visit.) Exclusion criteria are the same as for the New Tics group.

Inclusion for tic-free controls are: matched to children from the New Tics group on age (within 1 year), sex, handedness, and ADHD status. Exclusion criteria: current or past tic disorder in the subject or a first-degree relative, plus the exclusions listed for the New Tics group. We do not exclude OCD or ADHD; such exclusion would introduce another unwanted variable into the tics vs no-tics comparisons. However, both disorders are assessed thoroughly at screening.

### Clinical and Neuropsychological Measures

Data recorded includes demographics, brief IQ estimate (K-BIT II), the Child Behavior Checklist (CBCL), manual dexterity (Purdue Pegboard test) [50], attention (CPT II) [48], a “weather prediction” test of probabilistic classification kindly shared with us by Dr. Marsh [51,85], psychiatric comorbidity (DSM-5 diagnoses by K-SADS, confirmed by Dr. Black using all data), quantitative autistic traits (Social Responsiveness Scale [86]), quality of life (PedsQL; [87]), history of birth complications and maternal smoking during pregnancy, and family history. Dr. Black performs a neurological and psychiatric examination, reviews the parental self-report measures, rates current symptom severity (YGTSS [88], Children’s Yale-Brown Obsessive-Compulsive Scale (CY-BOCS) [89], ADHD Rating Scale [90]), and makes a final diagnosis of tic disorders, OCD, and ADHD using all data.

The following measures are also completed for subjects with tics: best estimate date of onset, typical features of TS according to the Diagnostic Confidence Index (DCI) [91], the Premonitory Urge for Tics Scale (PUTS) [92], and a standardized tic suppression paradigm (TSP) [47]. The TSP includes two 5-minute blocks of each of 3 conditions, seated alone in an exam room with a microphone and video camera: baseline (no tic suppression), verbal request to suppress tics, and differential reinforcement of zero-rate ticcing (DRO) using a remote-controlled token dispenser [82]. Video recordings are made of each 5-minute block to check scoring offline. The first 3 sessions are done completed in that order, then repeated in random order (that same order is maintained for future visits with that participant). More recently, we have prepended one 5-minute observation session with a staff member sitting quietly in the room with the participant, to measure social influence on tic suppression [93–95]. Children without tics will be similarly observed but will only complete the baseline condition (no tic suppression).

### MRI Methods

At the screening visit, we introduce each child to a mock scanner, which is equipped to give immediate feedback to the child on head motion. To enhance subject comfort and quality of scans, we use MRI acclimation materials adapted from the DirecNet MRI study of Type I diabetes, including an introductory video and a holding-still game to practice at home with a parent [96].

No caffeine intake is allowed before scans on study days. Subjects are monitored by video recording for offline identification of any visible tic during the 45 min of functional data collection. These data can be used to model or ignore fcMRI or ASL frames temporally related to the tics. Subjects who only partially complete adequate scans in the time allotted are invited to return, as soon as feasible, to complete the scans. We have adopted the FIRMM system to monitor head movement during scan sessions, allowing rapid assessment of whether more resting BOLD frames are needed [97].

We acquire 1 mm^3^ Tl-weighted MP-RAGE images with volumetric navigators (vNavs) for prospective motion correction [98], and T2-weighted images. BOLD data are from a whole-brain BOLD-sensitive asymmetric spin echo EPI sequence, during which participants are asked to rest quietly and watch a fixation crosshair. For perfusion imaging, we use a pulsed ASL sequence originally validated in children [99].

### Image Analysis

Using MP-RAGE structural MRI, we measure the volume and shape of the putamen, caudate, nucleus accumbens, globus pallidus, and thalamus using our published automated method involving high dimensional brain mapping [100]. We measure cortical thickness and volumes using Freesurfer software. In addition to these tests of *a priori* hypotheses, we plan a 3D analysis using standard voxel-based morphometry (VBM) methods that we have used previously [56,101–103].

BOLD pre-processing and rs-fcMRI analyses are carried out as previously described [64,71,104–106]. We use very stringent criteria for BOLD fMRI data quality [69], since even small-amplitude head motion can cause spurious changes in rs-fcMRI analysis [67,107]. Quantitative measures of frame-to-frame head displacement (FD) are used to reduce motion artifact using the following criteria: we will include BOLD-sensitive data volumes only in temporally contiguous sets of at least 5 volumes with framewise displacement <0.2 mm each, including BOLD runs only if they retained a minimum of 30 such volumes. Justification of these parameters, which may change with improved methodological research, appears with additional technical details in ref. [71]. We will similarly remove tic-related activity (using the audiovisual recordings acquired during scans). Next, we extract resting state time series from seed ROIs, correlate the BOLD time series region by region [108], combine correlation coefficients (*r*) across participants [109,110], and test whether correlation coefficients are significantly different from zero (2-tailed *t* tests, corrected). Interregional correlations are measured over different thresholds and using network measures (e.g., participation coefficient, local clustering). Group differences will be analyzed using graph analysis tools [111,112], including quantitative (network modularity [113]) and qualitative methods (Social Network Image Animator [114] with spring embedding algorithms as in ref. [115]).

### Statistical Analysis

The primary approach to analyzing each main hypothesis is a general linear model; we include nuisance variables if they explain a significant portion of the total variance (e.g., total intracranial volume, age, sex, and handedness). The independent variable was originally planned to be change in YGTSS total tic score from screening to follow-up (“ΔTTS”); we have since focused on follow-up TTS score as being more clinically meaningful, and control for baseline TTS score.

We will also perform multivariate analyses to account for the large quantities of high-dimensional data using support vector machine (SVM) classification. SVMs are algorithms that take a training set of labeled samples, each with a series of measurements called features, and learn properties and weights of the features that characterize the labels. Once a decision function is learned based on the training data, it can be used to predict the class label of a new, previously unseen, test sample. Support Vector Regression (SVR) extends SVM to dimensional regression in order to predict a continuous measure for an individual, rather than a class label. Here we apply methods previously used in our lab [77,81,116–118]. See [119–122] for mathematical descriptions of SVMs. We use the Spider Machine Learning Toolbox for computations [123]. We will use SVM in several ways. First, we will apply it in Aim 1 as a method primarily for exploratory analysis. In this case the training data will include all subjects in each of the two groups being compared. An estimate of reliability of accuracies can be computed testing on the same data set using a leave-one-out cross validation approach, as previously described [71,81]. We hypothesize that classification accuracy will be higher for an SVM based on the full data set (clinical data, structural MRI, perfusion MRI, tic-triggered fMRI, and rs-fcMRI data), in which the features will include assessment scores, regional volumes, voxelwise rCBF, tic-related BOLD activity, and interregional temporal correlations, than for an SVM based on one data type alone. Similarly, we will use SVR in Aim 2 as an exploratory approach to identify patterns in the rich clinical and MRI data set that best predict follow-up symptom severity (TTS), rather than categorical outcome.

We will also use SVM as a primary approach to identifying features that predict which New Tics subjects will progress to TS/CTD (or SVR, to predict who will progress to more *vs.* less symptomatic). This approach will *train* the SVM on the Existing TS/CTD and Tic-free Control data sets, and then *test* baseline data from individual New Tics subjects to “diagnose” them as more like Existing TS/CTD or more like Tic-free Control subjects. The hypothesis is that this SVM approach will identify significantly better than chance which New Tics subjects will remit (or become asymptomatic) and which will be diagnosed with TS/CTD (or remain symptomatic).

## RESULTS

### Study Progress

#### Recruitment sources and follow-up success

In the New Tics group, 33% of participants came from clinical referrals, while 54% were recruited by advertising (via local school districts, online, or in traditional media).

Of the 82 New Tics participants who have completed a first visit, 65 have returned for the 12-month follow-up visit and an additional 9 are still awaiting return visits, leaving only 8 (9.6%) who dropped out or were lost to follow-up. After the first 44 participants, we began to invite New Tics subjects to return at 24, 36 or 48 months after their first tic. As of April, 2020, we have completed 31 24-month, 10 36-month and 6 48-month visits.

A number of other children were brought in by parents who originally reported a tic duration of only a few months, but definite tics more than a year ago were discovered on review of history or on examination, giving them a DSM-5 diagnosis of Tourette syndrome. These children were invited to participate in the Established TS/CTD group. For 9 children, the baseline tic duration fell in the range of 9–11.5 months; these few are being followed under separate funding, with a first follow-up visit scheduled 3 months after the baseline visit.

#### Sample characteristics

**Figure 2.**
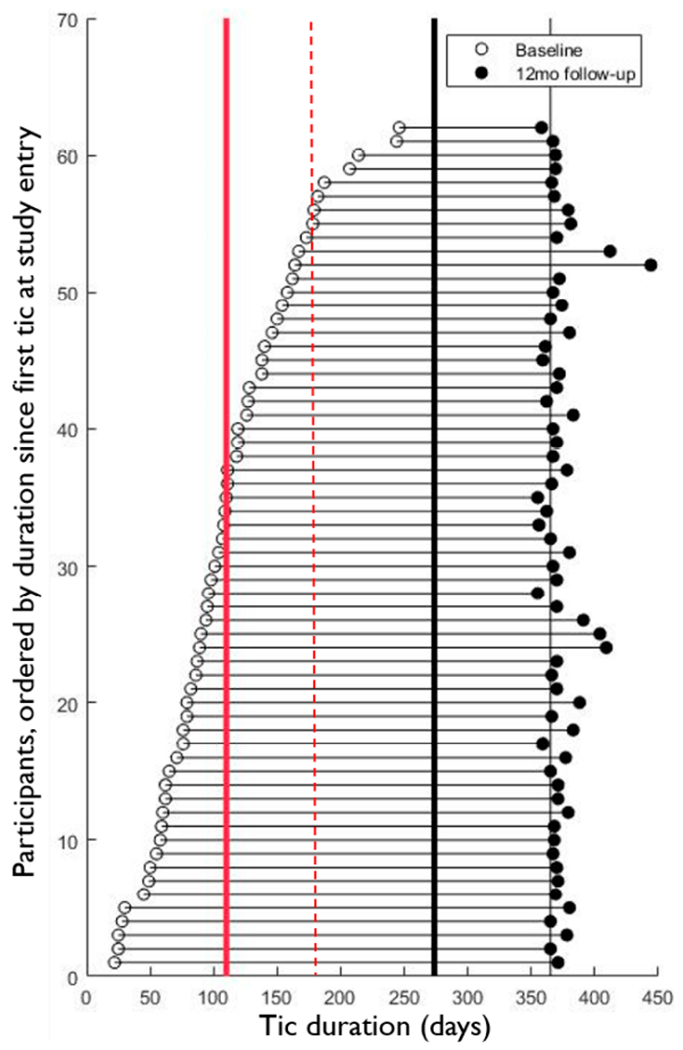
Tic duration at study entry and follow-up visits. The horizontal axis represents days since first tic. Individual participants are shown as horizontal lines, with their first study visit marked by an open circle and their 12-month follow-up visit marked by a filled circle. The solid red line marks the current mean duration at the baseline study visit, the dashed red line marks the original 6-month cutoff for enrollment, the thick black line marks the current 9-month cutoff, and the thin black vertical line marks 1 year after the first tic.

Vigorous recruitment efforts have led to a sample that on average has relatively mild symptoms and was seen within a few months of their first tic (see Table 1, which includes only the children who have already completed a follow-up visit). However, the range of severity is wide, and the distribution of time since onset of tics is fairly smooth (Figure 2). Most of the children have experienced several motor tics and at least one phonic tic by the baseline visit (though some of these were not previously identified by the children or their parents).

At the follow-up visit, on average tic severity has improved, though often other tics have appeared in the interim (Table 2). Other classic TS features have also often appeared, such as premonitory urges or intentional suppression, so DCI score generally has increased.

#### MRI data quality

Neuroimaging studies in clinical samples of children often have relatively high rates of data loss [126], and we knew that collecting quality rs-fcMRI data would be difficult in children with tics as young as age 5, many with ADHD. Our initial efforts reflected that concern, with adequate quality data for rs-fcMRI analysis in only 3 of the first 14 subjects enrolled. Therefore, we consulted with colleagues and implemented several steps to improve quality. These included more extensive subject preparation [96], training in a mock MRI scanner with immediate head movement feedback, and allowance for a possible second scan day. Since implementing these changes, we have collected adequate rs-fcMRI data in 34 of the remaining 47 subjects scanned (72%). For the structural MRI scans, we have high quality images (based on standardized manual ratings) from 41 of 54 children scanned (76%).

### Reward Enhances Tic Suppression in Children with PTD

Our first publication from this project came from the first 21 children enrolled [82]. That report discusses in detail our decisions about dating the first tic. Tic suppression was well known in TS/CTD, and was known to improve in the presence of an immediate, contingent reward for periods with no tics (differential reinforcement of other, DRO). However, given how TS/CTD were defined, children in those previous studies had experienced tics for at least a year, so whether tic inhibition was present initially or learned over time was not known. Results showed that these children, studied on average 3.5 months after onset of tics, generally could suppress tics, especially in the DRO condition. More than half suppressed tics (i.e., had more 10-s tic-free periods) with a verbal request to suppress tics, or with a control condition in which rewards were given regardless of whether the child was ticcing (noncontingent reward, NCR). All but 3 of them suppressed tics during the DRO condition. Time since tic onset did not correlate significantly with suppression ability. In other words, these children, like children with TS/CTD, do have some capacity to suppress tics, and immediate reward enhances that capacity.

Data from that report also contributed substantially to a multi-site summary of individual participant data on voluntary tic suppression in children with PTD or TS/CTD [127]. Since then, we have confirmed the key findings from that initial report in a later superset of patients [128].

### Transient Tics Aren’t

DSM-IV and DSM-IV-TR diagnosed children who had been ticcing for less than a year with Transient Tic Disorder. Despite the name, tics in these children did not always prove to be transient, as every child with TS/CTD originally had tics for less than a year. (This observation was one motivation for creating the PTD diagnosis in DSM-5; see discussion in ref. [6]). As reviewed above, professional consensus had been that these tics would almost always disappear, though the limited available data did not confirm that consensus.

We reported clinical outcomes for the New Tics children enrolled in our preliminary data [124]. These first 43 children comprise the largest ever prospective study of PTD. At the baseline visit, although tics were relatively mild for most children (only 9 showed a TTS > 20, indicating moderate or severe tic severity), the symptoms generally concerned parents; 12 of 15 families who were asked reported they had taken or would be taking their child to a doctor because of the tics (this question was added midway through the study).

Thirty-nine of the 43 (90%) returned for the 12-month follow-up visit. To our surprise, every child still had tics. Tics were observed at the visit in all but one child, sometimes only when observed alone (by audio-video feed) during the tic suppression paradigm. The remaining child had tics reported by parents in the past week, and was observed to tic on a later encounter. Thus at follow-up, the DSM-5 diagnosis was Persistent (Chronic) Motor Tic Disorder in 3 children and Tourette’s Disorder in all the rest. DCI scores had increased by a mean of 9 points, showing gradual accumulation of more classic TS features. These results call into doubt the general assumption of complete remission for PTD. Even if all 4 of the participants lost to follow-up remitted completely, at least 90% (39/43) of the total sample still showed tics at the one-year anniversary of tic onset. That fraction implies 95% confidence that the population remission rate at 12 months is <22%.

On the other hand, on average tics had improved. Only six participants met DSM-IV criteria for Tourette’s Disorder, which required impairment in a life role or marked distress, and only two had mild or greater impairment in a life role. We asked 29 parents if they were planning to see a doctor for further management of their child’s tics, and only 3 said yes. In fact, several children and parents felt the tics were gone. In some cases (at least 9 of 28), no tics were seen at the 12-month visit during a typical neurological history and physical exam supplemented by information needed for YGTSS rating. In routine clinical practice, these children would have been considered remitted.

In a practical sense, the news is still good for these families, as the tics were only clinically problematic at follow-up in 5% (mild impairment or more) to 10% (planning to seek further clinical care). The difference from previous clinical consensus appears most likely to be in the fact that many of these patients would not be seeing a doctor for clinical follow-up, and in the thoroughness of the history and exam at follow-up.

An alternative explanation could be that tics usually do completely remit, but after the 1-year mark. To begin to explore that possibility, we are continuing the planned 24-, 36- and 48-month follow-up visits. Thus far, almost every such visit has continued to show evidence of tics, though they have apparently completely remitted in a small number of cases. We look forward to completing these visits in a few years.

This report also identified several clinical features at the baseline visit that predicted better tic outcome at follow-up. Not surprisingly, baseline TTS was correlated with TTS at follow-up, so additional analyses controlled for baseline severity. Worse outcomes were predicted by the following features at baseline: higher Social Responsiveness Scale (SRS) score, higher DCI score, higher CBCL-DESR score, the presence of an anxiety disorder, and a history of 3 or more phonic tics. In a stepwise multiple regression analysis, a model with TTS, SRS score, and anxiety disorder, at baseline, explained nearly half the variance in the 12-month TTS score. An elevated SRS score indicates a greater number of features associated with autism spectrum disorder, though in this sample all the SRS scores were in the normal range.

### Tic Suppression Ability Predicts Outcome

We had hypothesized that better tic suppression would predict better clinical outcomes [82]. Last year, we reported a test of this hypothesis on the first 45 children to complete their 12-month visits [128]. Those children with better tic suppression in the presence of a reward had lower tic burden (TTS scores) at the 12-month follow-up visit. Additionally, children with more premonitory urges tended to suppress tics more successfully. This report identifies another potential predictor of clinical outcome in PTD and suggests one mechanism that may help reconcile the persistence of tics, with careful observation when the child is alone, with the improvement in clinical status. Specifically, clinical improvement is driven in large part by less frequent or less severe tics when around other people, and perhaps the experience of social rewards for tic suppression lead over time to (perhaps unintentional) tic suppression around others. To begin to measure social influence on tic suppression, recently we have added a session to the tic suppression protocol. A staff member sits with the child without engaging in conversation and without any explicit direction to suppress tics; this setting was shown to decrease tic expression compared to the child sitting alone [93–95].

### Other Behavioral Measures

In the first 58 children to complete the 1-year follow-up visit, we tested the predictive value of several other cognitive or motor tasks we examined based on past studies in TS/CTD, namely the Purdue Pegboard Test (measuring dexterity and bimanual coordination), the Continuous Performance Test (measuring attention and response inhibition), and a weather prediction task (intended to quantify a form of habit learning). None significantly predicted 1-year tic outcome, and none correlated significantly with tic suppression ability after controlling for age [129].

### Subcortical Volumetry

One of our primary hypotheses was that caudate volume would correlate inversely with tic severity at follow-up. We recently submitted for publication results from testing this hypothesis in the 41 children with good-quality structural MRI data at baseline and had completed the follow-up visit by December, 2019 [130]. We used a method that simultaneously and reliably quantified the volume of several subcortical structures. Caudate volume was not a significant prognostic factor. However, a larger hippocampus at baseline predicted higher tic severity at 12-month follow-up (*p* = 0.002). Notably, this result was confirmed in a subgroup of 25 children whose MRI was performed using prospective motion correction to minimize artifactual changes to regional brain volumes induced by head movement during the scan. In this group, baseline hippocampal volume and baseline TTS explained over half the variance in 12-month TTS score. Although we did not include hippocampal volume in our hypotheses for this project, previous studies have identified larger hippocampal volume in children with TS [131], and both the PTD and TS groups tended to have greater volume in our study as well. The hippocampus is involved in memory consolidation for motor habit learning as well as declarative learning [132], and in youth with TS, there is evidence that those with more severe tics have more persistent motor memory, in that they take longer to unlearn a previously learned pattern of behavior [133]. These observations allow the hypothesis that greater hippocampal volume may mediate relatively inflexible habit learning, leading to greater persistence of tics between the initial assessment and the 1-year mark.

### Other Contributions

As part of our efforts to recruit children early in the course of PTD, we undertook an observational study at a local elementary school, similar to a previous study by Snider and colleagues [17]. Experienced raters including authors KJB and DJG observed various classrooms for at least an hour, noting children with probable tics observed at least 3 times in the hour. These children were approximately 5–11 years old. Ratings were done on visits to the school in each of 3 months (March, April and May). Initial results suggest that tics were noted in 10–25% of children, depending on the month, roughly consistent with the mean cross-sectional tic frequency of 24% noted by Snider et al.

We developed software to facilitate recording of the timing of tics during the TSP, and to automate delivery of rewards under the DRO and NCR conditions, and have made it freely available [134]. Some of the participants in this study signed a separate consent to let their video recordings be used for the “VISIT-TS,” which is a video-enhanced screening instrument to ask people if they (or their children) have tics after showing them a 5-minute video with nearly 100 video clips of tics [135].

## DISCUSSION

Many children have tics at least briefly, whereas few are diagnosed with a chronic tic disorder. This observation inspired the present project, in which the disappearance (or improvement) of tics can be observed prospectively. Most previous studies on PTD were retrospective, and most enrolled children in a clinical setting, introducing bias for severity and treatment availability. However, understanding why tics usually improve over the first year after their appearance may provide clues to alleviating chronic tics.

Thus far, with vigorous outreach and advertising, we have maintained adequate recruitment of children with recent onset of tics (mean <4 months), a population that has been difficult to enroll. We confirmed that, like children with a chronic tic disorder, tic suppression is possible within the first few months after tic onset and is improved by providing immediate, contingent rewards for successful tic suppression. More important and more surprising, we have demonstrated that the course of PTD is almost always one of improvement rather than disappearance of symptoms at the 12-month mark that defines chronicity in the current nosology. We have further identified several indicators at initial enrollment that predict worse or better clinical tic status at 12 months. Some of these are clinical measures that are relatively easily assessed, including baseline tic severity and variety, subsyndromal autistic features, and anxiety. Better intentional tic suppression under conditions of immediate reward proved to be another relatively straightforward indicator of improvement at follow-up. Finally, we identified hippocampal volume as a novel prognostic biomarker over the first year of tic symptoms. All of these are novel results with potential clinical relevance. The project continues enrollment, but we have reported important, clinically relevant results from what is already the largest prospective study of PTD.

The primary limitation of our work is that our sample was not a purely representative epidemiological sample. Such a sample would be extremely difficult to study, since a relatively large number of children would need to be thoroughly screened, and since most would be asymptomatic, limiting child and parent enthusiasm for participation. The most obvious concern related to representativeness is that we may be likely to oversample children with severe tics or from families better positioned to access medical care. However, we feel that our sample is fairly representative. Tic severity was fairly low at study entry, with a mean TTS < 20; about a third of the children came from families experienced with tics (positive family history or a physician parent); and disadvantaged minorities are represented at or above the frequency predicted from regional demographics.

Remaining work includes first completing enrollment of the full sample, which was predicted to provide adequate power for the remaining analyses, especially rs-fcMRI, voxel-based morphometry, perfusion fMRI, and the machine learning analyses. Other planned analyses include a shape deformation analysis of basal ganglia, thalamus and hippocampus by our colleague Dr. Lei Wang from Northwestern University [100,136,137]. We are also eager to find out how often tics completely remit by 2, 3 and 4 years after tic disorder onset.

One possible future direction may be to test whether it is possible to effectively reduce future tic severity by a randomized controlled trial of behavior therapy in the New Tics children most likely to need continuing clinical attention. Potentially this approach would produce secondary prevention of TS/CTD. The prognostic indicators we have identified will allow directing treatment to those children most likely to benefit from intervention, a resource allocation benefit in this population with relatively good outcomes overall. The best proven behavioral treatment for chronic tic disorders is CBIT, but alternatives that may lend themselves well to children of this age, early in the course of illness, include CBIT-Jr, which focuses more on family accommodation and attention to tics [138], or exposure and response prevention (ERP) [139]. For ERP we have proposed a modification, based on the tic suppression results described above, that may facilitate participation by young children [140]. Alternatively, perhaps a cognitive remediation strategy could focus on improving the ability to unlearn habitual maladaptive motor sequences. Of course, novel results after completion of enrollment may point us in different directions.

## Figures and Tables

**Table 1.** Participant characteristics at baseline among those who completed a 12-month visit. Values indicate number or mean ± SD unless indicated otherwise.

Sample characteristic	Baseline
*N*	65
Age	7.81 ± 2.03
Sex (M:F)	44:21
Handedness (R:not R)	57:8
Non-white (*N* = 63)	5
Barratt SES	52.03 ± 10.52 ^1^
IQ estimate (K-BIT)	108.94 ± 12.31
Non-tic K-SADS diagnosis	53
ADHD	23
OCD	6
Anxiety disorder	33
Medication	12 ^2^
SRS total T scores	49.72 ± 9.48

1 Highest possible score 66.

2 Includes only daily or more frequent brain-active medications.

**Table 2.** Change in tic characteristics among those who completed a 12-month visit. Values in parentheses indicate mean ± SD unless indicated otherwise.

Sample characteristic	Baseline	12-month
Days since tic onset	112.28 ± 56.44	374.17 ± 16.51
YGTSS total	24.72 ± 12.33	17.92 ± 12.83
Total tic score	17.06 ± 5.89	13.62 ± 7.39
Motor	10.55 ± 3.89	8.91 ± 4.57
Phonic	6.49 ± 4.50	4.71 ± 4.19
Impairment	7.66 ± 8.00	4.31 ± 6.84
DCI score	32.63 ± 13.01	43.14 ± 15.36
Ever had a phonic tic	53	56
Ever had a complex tic	30	41
DSM-IV TS or CTD	0	21
DSM-IV-TR TS or CTD	0	58^1^
DSM-5 TS or CTD	0	64^2^

1 The other 7 children had a reported interim remission of 3 months or more, for a diagnosis of a repeated episode of Transient Tic Disorder. See discussion in ref. [124].

2 The remaining child had experienced several phonic tics but only one motor tic [125].

## ABBREVIATIONS

TS: Tourette syndrome
CTD: Persistent (Chronic) Motor or Vocal Tic Disorder
PTD: Provisional Tic Disorder
CBIT: Comprehensive Behavioral Intervention for Tics
NIH: National Institutes of Health
YGTSS: Yale Global Tic Severity Scale
TTS: total tic score from the YGTSS
MRI: magnetic resonance imaging
fMRI: functional MRI
fcMRI: functional connectivity fMRI
rs-fcMRI: resting state functional connectivity fMRI
CPT: Continuous Performance Test
DCI: Diagnostic Confidence Index
ASL: arterial spin labeling perfusion fMRI
BOLD: blood oxygen level–dependent fMRI signal
VBM: voxel-based morphometry
SVM: support vector machine
SVR: support vector regression
DRO: differential reinforcement of other behavior
NCR: noncontingent reward
SRS: Social Responsiveness Scale
CBCL: Child Behavior Checklist
DESR: deficient emotional self-regulation
ERP: exposure and response prevention

## References

[1] Black KJ. Tics In: Kompoliti K, Verhagen Metman L, Cornelia C, Goetz C, Goldman J, Kordower J, , editors. Encyclopedia of Movement Disorders. Oxford (UK): Elsevier (Academic Press); 2010 p. 231–6.

[2] Schlaggar BL, Mink JW. Movement disorders in children. Pediatr Rev. 2003;24(2):39–51. 12563038 10.1542/pir.24-2-39

[3] Leckman JF, Zhang H, Vitale A, Lahnin F, Lynch K, Bondi C, Course of tic severity in Tourette syndrome: the first two decades. Pediatrics. 1998;102(1 Pt 1):14–9. 9651407 10.1542/peds.102.1.14

[4] The Tourette Syndrome Classification Study Group. Definitions and classification of tic disorders. Arch Neurol. 1993;50(10):1013–6. 8215958 10.1001/archneur.1993.00540100012008

[5] American Psychiatric Association. Diagnostic and Statistical Manual of Mental Disorders, Fifth Edition Arlington (VA, USA): American Psychiatric Association; 2013.

[6] Black KJ, Black ER, Greene DJ, Schlaggar BL. Provisional Tic Disorder: What to tell parents when their child first starts ticcing [version 1]. F1000Res. 2016;5:696. 27158458 10.12688/f1000research.8428.1 PMC4850871

[7] Leckman JF, Bloch MH, Smith ME, Larabi D, Hampson M. Neurobiological substrates of Tourette’s disorder. J Child Adolesc Psychopharmacol. 2010;20(4):237–47. 20807062 10.1089/cap.2009.0118 PMC2958453

[8] Singer HS. Tourette syndrome and other tic disorders. Handb Clin Neurol. 2011;100:641–57. 21496613 10.1016/B978-0-444-52014-2.00046-X

[9] Greene DJ, Black KJ, Schlaggar BL. Neurobiology and functional anatomy of tic disorders In: Martino D, Leckman JF, editors. Tourette Syndrome. Oxford (UK): Oxford University Press; 2013.

[10] Cuenca J, Glazebrook C, Kendal T, Hedderly T, Heyman I, Jackson G, Perceptions of treatment for tics among young people with Tourette syndrome and their parents: a mixed methods study. BMC Psychiatry. 2015;15:46. 25879205 10.1186/s12888-015-0430-0 PMC4359496

[11] Robertson MM. The prevalence and epidemiology of Gilles de la Tourette syndrome. Part 1: the epidemiological and prevalence studies. J Psychosom Res. 2008;65(5):461–72. 18940377 10.1016/j.jpsychores.2008.03.006

[12] Mason A, Banerjee S, Eapen V, Zeitlin H, Robertson MM. The prevalence of Tourette syndrome in a mainstream school population. Dev Med Child Neurol. 1998;40(5):292–6. 9630255

[13] Kurlan R, Como PG, Miller B, Palumbo D, Deeley C, Andresen EM, The behavioral spectrum of tic disorders: a community-based study. Neurology. 2002;59(3):414–20. 12177376 10.1212/wnl.59.3.414

[14] Hornsey H, Banerjee S, Zeitlin H, Robertson M. The prevalence of Tourette syndrome in 13–14-year-olds in mainstream schools. J Child Psychol Psychiatry. 2001;42(8):1035–9. 11806685 10.1111/1469-7610.00802

[15] Peterson BS, Pine DS, Cohen P, Brook JS. Prospective, longitudinal study of tic, obsessive-compulsive, and attention-deficit/hyperactivity disorders in an epidemiological sample. J Am Acad Child Adolesc Psychiatry. 2001;40(6):685–95. 11392347 10.1097/00004583-200106000-00014

[16] Cubo E, Gabriel y Galan JMT, Villaverde VA, Velasco SS, Benito VD, Macarron JV, Prevalence of tics in schoolchildren in central Spain: a population-based study. Pediatr Neurol. 2011;45(2):100–8. 21763950 10.1016/j.pediatrneurol.2011.03.003

[17] Snider LA, Seligman LD, Ketchen BR, Levitt SJ, Bates LR, Garvey MA, Tics and problem behaviors in schoolchildren: prevalence, characterization, and associations. Pediatrics. 2002;110(2 Pt 1):331–6. 12165586 10.1542/peds.110.2.331

[18] Dooley JM. Tic disorders in childhood. Semin Pediatr Neurol. 2006;13(4):231–42. 17178353 10.1016/j.spen.2006.09.004

[19] Tics Kuperman S. and Tourette’s syndrome in childhood. Semin Pediatr Neurol. 2003;10(1):35–40. 12785746 10.1016/s1071-9091(02)00007-4

[20] Bloch MH, Leckman JF. Tic disorders In: Martin A, Volkmar FR, Lewis M, editors. Lewis’s Child and Adolescent Psychiatry: A Comprehensive Textbook. 4th ed. Philadelphia (PA,US): Lippincott Williams & Wilkins; 2007 p. 569–82.

[21] Zinner SH, Mink JW. Movement disorders I: tics and stereotypies. Pediatr Rev. 2010;31(6):223–33. 20516234 10.1542/pir.31-6-223

[22] Jung HY, Chung SJ, Hwang JM. Tic disorders in children with frequent eye blinking. J AAPOS. 2004;8(2):171–4. 15088052 10.1016/j.jaapos.2003.10.007

[23] Scahill L, Sukhodolsky DG, Williams SK, Leckman JF. Public health significance of tic disorders in children and adolescents. Adv Neurol. 2005;96:240–8. 16383223

[24] Shapiro AK, Shapiro E, Young JG, Feinberg TE. Gilles de la Tourette Syndrome. 2nd ed. New York (US): Raven Press; 1988 p. 171.

[25] Fourneret P, Desombre H, Broussolle E. [From tic disorders to Tourette syndrome: current data, comorbidities, and therapeutic approach in children]. Arch Pediatr. 2014;21(6):646–51. 24815597 10.1016/j.arcped.2014.03.017

[26] Bruun RD, Budman CL. The course and prognosis of Tourette syndrome. Neurol Clin. 1997;15(2):291–8. 9115462 10.1016/s0733-8619(05)70313-3

[27] Carter AS, Pauls DL, Leckman JF, Cohen DJ. A prospective longitudinal study of Gilles de la Tourette’s syndrome. J Am Acad Child Adolesc Psychiatry. 1994;33(3):377–85. 8169183 10.1097/00004583-199403000-00012

[28] Spencer T, Biederman M, Coffey B, Geller D, Wilens T, Faraone S. The 4-year course of tic disorders in boys with attention-deficit/hyperactivity disorder. Arch Gen Psychiatry. 1999;56(9):842–7. 12884890 10.1001/archpsyc.56.9.842

[29] Hong KE. A clinical study of tic disorder in Korea. J Korean Pediatr Assoc. 1981;24(3):198–208.

[30] Shin ZH, Jung C-H, Kim HC. Follow-up study of the tic disorders. J Korean Acad Child Adolesc Psychiatry. 1996;7(1):68–76.

[31] Shapiro E, Shapiro AK. Semiology, nosology and criteria for tic disorders. Rev Neurol (Paris). 1986;142(11):824–32. 3469716

[32] Goetz CG, Tanner CM, Stebbins GT, Leipzig G, Carr WC. Adult tics in Gilles de la Tourette’s syndrome: description and risk factors. Neurology. 1992;42(4):784–8. 1565232 10.1212/wnl.42.4.784

[33] de Groot CM, Bornstein RA, Spetie L, Burriss B. The course of tics in Tourette syndrome: a 5-year follow-up study. Ann Clin Psychiatry. 1994;6(4):227–33. 7647832 10.3109/10401239409149009

[34] Pappert EJ, Goetz CG, Louis ED, Blasucci L, Leurgans S. Objective assessments of longitudinal outcome in Gilles de la Tourette’s syndrome. Neurology. 2003;61(7):936–40. 14557563 10.1212/01.wnl.0000086370.10186.7c

[35] Coffey BJ, Biederman J, Geller D, Frazier J, Spencer T, Doyle R, Reexamining tic persistence and tic-associated impairment in Tourette’s Disorder: Findings from a naturalistic follow-up study. J Nerv Ment Dis. 2004;192(11):776–80. 15505522 10.1097/01.nmd.0000144696.14555.c4

[36] Bloch MH, Leckman JF, Zhu H, Peterson BS. Caudate volumes in childhood predict symptom severity in adults with Tourette syndrome. Neurology. 2005;65(8):1253–8. 16247053 10.1212/01.wnl.0000180957.98702.69 PMC2367161

[37] Bloch ΜΗ, Peterson BS, Scahill L, Otka J, Katsovich L, Zhang H, Adulthood outcome of tic and obsessive-compulsive symptom severity in children with Tourette syndrome. Arch Pediatr Adolesc Med. 2006;160(1):65–9. 16389213 10.1001/archpedi.160.1.65 PMC2291298

[38] Leckman JF, Bloch MH, King RA, Scahill L. Phenomenology of tics and natural history of tic disorders. Adv Neurol. 2006;99:1–16. 16536348

[39] Cavanna AE, David K, Orth M, Robertson MM. Predictors during childhood of future health-related quality of life in adults with Gilles de la Tourette syndrome. Eur J Paediatr Neurol. 2012;16(6):605–12. 22381812 10.1016/j.ejpn.2012.02.004

[40] Groth C Tourette syndrome in a longitudinal perspective. Clinical course of tics and comorbidities, coexisting psychopathologies, phenotypes and predictors. Dan Med J. 2018;65(4):B5465. 29619935

[41] Bäumer T, Sajin V, Munchau A. Childhood-onset movement disorders: A clinical series of 606 cases. Mov Disord Clin Pract. 2017;4(3):437–40. 30363430 10.1002/mdc3.12399 PMC6174494

[42] Peterson BS, Riddle MA, Cohen DJ, Katz LD, Smith JC, Hardin MT, Reduced basal ganglia volumes in Tourette’s syndrome using three-dimensional reconstruction techniques from magnetic resonance images. Neurology. 1993(43):941–9. 8492950 10.1212/wnl.43.5.941

[43] Plessen KJ, Bansal R, Peterson BS. Imaging evidence for anatomical disturbances and neuroplastic compensation in persons with Tourette syndrome. J Psychosom Res. 2009;67(6):559–73. 19913660 10.1016/j.jpsychores.2009.07.005 PMC4283588

[44] Black KJ. Behavior therapy for Tourette syndrome and other tic disorders. 2017 Available from: https://tics.wustl.edu/treatment/behavior-therapy-for-tics/. Accessed 2020 May 27.

[45] Khalifa N, von Knorring AL. Tourette syndrome and other tic disorders in a total population of children: clinical assessment and background. Acta Paediatr. 2005;94(11):1608–14. 16352498 10.1111/j.1651-2227.2005.tb01837.x

[46] Greene DJ, Koller JM, Schlaggar BL, Black KJ. “Can you stop that?” Ability to suppress tics is present within months of tic onset, and can predict future clinical outcome. Present at Annual meeting, Society for Neuroscience; 2012 Oct 13-17; New Orleans, LA, USA Poster #764.01.

[47] Woods DW, Himle MB. Creating tic suppression: comparing the effects of verbal instruction to differential reinforcement. J Appl Behav Anal. 2004;37(3):417–20. 15529900 10.1901/jaba.2004.37-417 PMC1284518

[48] Woods DW, Himle MB, Miltenberger RG, Carr JE, Osmon DC, Karsten AM, Durability, negative impact, and neuropsychological predictors of tic suppression in children with chronic tic disorder. J Abnorm Child Psychol. 2008;36(2):237–45. doi: 10.1007/s10802-007-9173-9 17717739

[49] Chang HL, Liang HY, Wang HS, Li CS, Ko NC, Hsu YP. Behavioral and emotional problems in adolescents with Tourette syndrome. Chang Gung Med J. 2008;31(2):145–52. 18567414

[50] Bloch MH, Sukhodolsky DG, Leckman JF, Schultz RT. Fine-motor skill deficits in childhood predict adulthood tic severity and global psychosocial functioning in Tourette’s syndrome. J Child Psychol Psychiatry. 2006;47(6):551–9. 16712631 10.1111/j.1469-7610.2005.01561.x

[51] Marsh R, Alexander GM, Packard MG, Zhu H, Wingard JC, Quackenbush G, Habit learning in Tourette syndrome: a translational neuroscience approach to a developmental psychopathology. Arch Gen Psychiatry. 2004;61(12):1259–68. 15583117 10.1001/archpsyc.61.12.1259

[52] Kurlan R, Lichter D, Hewitt D. Sensory tics in Tourette’s syndrome. Neurology. 1989;39(5):731–4. 2710364 10.1212/wnl.39.5.731

[53] Rozenman M, Johnson OE, Chang SW, Woods DW, Walkup JT, Wilhelm S, Relationships between premonitory urge and anxiety in youth with chronic tic disorders. Child Health Care. 2015;44(3):235–48. 27110050 10.1080/02739615.2014.986328 PMC4840885

[54] Draper A, Jackson GM, Morgan PS, Jackson SR. Premonitory urges are associated with decreased grey matter thickness within the insula and sensorimotor cortex in young people with Tourette syndrome. J Neuropsychol. 2016;10(l):143–53. 26538289 10.1111/jnp.12089 PMC4982075

[55] McGuire JF, Piacentini J, Scahill L, Woods DW, Villarreal R, Wilhelm S, Bothersome tics in patients with chronic tic disorders: Characteristics and individualized treatment response to behavior therapy. Behav Res Ther. 2015;70:56–63. 25988365 10.1016/j.brat.2015.05.006 PMC4449823

[56] Greene DJ, Williams AC III, Roller JM, Schlaggar BL, Black KJ, The Tourette Association of America Neuroimaging Consortium. Brain structure in pediatric Tourette syndrome. Mol Psychiatry. 2017;22(7):972–80. 27777415 10.1038/mp.2016.194 PMC5405013

[57] Greene DJ, Kim S, Black KJ, Schlaggar BL. Neurobiology and functional anatomy of tic disorders In: Martino D, Leckman JF, editors. Tourette Syndrome. 2nd ed. Oxford (UK): Oxford University Press; 2020 in press.

[58] Diler RS, Reyhanli M, Toros F, Kibar M, Avci A. Tc-99m-ECD SPECT brain imaging in children with Tourette’s syndrome. Yonsei Med J. 2002;43(4):403–10. 12243130 10.3349/ymj.2002.43.4.403

[59] Moriarty J, Costa DC, Schmitz B, Trimble MR, Ell PJ, Robertson MM. Brain perfusion abnormalities in Gilles de la Tourette’s syndrome. Br J Psychiatry. 1995;167:249–54. 7582678 10.1192/bjp.167.2.249

[60] Black KJ, Roller JM, Campbell MC, Gusnard DA, Bandak SI. Quantification of indirect pathway inhibition by the adenosine A2a antagonist SYN115 in Parkinson disease. J Neurosci. 2010;30(48):16284–92. 21123574 10.1523/JNEUROSCI.2590-10.2010 PMC3008651

[61] Black KJ, Duvall LB, Campbell MC, Roller JM. Signal and noise in continuous arterial spin labeling (cASL) as a function of time. 2008 [updated 2009/12/17/]. Available from: http://www.abstractsonline.com/Plan/ViewAbstract.aspx?sKey=a9936bf9-50db-482c-8a46-d6059322deda&cKey=0a4a298e-5775-4248-bc5c-le7cl25489ef. Accessed 2020 May 27.

[62] Stewart SB, Roller JM, Campbell MC, Black KJ. Arterial spin labeling versus BOLD in direct challenge and drug-task interaction pharmacological fMRI. PeerJ. 2014;2:e687. 25538867 10.7717/peerj.687 PMC4266850

[63] Stewart SB, Roller JM, Campbell MC, Perlmutter JS, Black KJ. Additive global cerebral blood flow normalization in arterial spin labeling perfusion imaging. PeerJ. 2015;3:e834. 25802806 10.7717/peerj.834 PMC4369335

[64] Church JA, Fair DA, Dosenbach NU, Cohen AL, Miezin FM, Petersen SE, Control networks in paediatric Tourette syndrome show immature and anomalous patterns of functional connectivity. Brain. 2009;132(Pt 1):225–38. 18952678 10.1093/brain/awn223 PMC2638693

[65] Church JA, Wenger KK, Dosenbach NU, Miezin FM, Petersen SE, Schlaggar BL. Task control signals in pediatric Tourette syndrome show evidence of immature and anomalous functional activity. Front Hum Neurosci. 2009;3:38. 19949483 10.3389/neuro.09.038.2009 PMC2784679

[66] Fair DA, Cohen AL, Dosenbach NU, Church JA, Miezin FM, Barch DM, The maturing architecture of the brain’s default network. Proc Natl Acad Sci U S A. 2008;105(10):4028–32. 18322013 10.1073/pnas.0800376105 PMC2268790

[67] Power JD, Barnes KA, Snyder AZ, Schlaggar BL, Petersen SE. Spurious but systematic correlations in functional connectivity MRI networks arise from subject motion. Neuroimage. 2012;59(3):2142–54. 22019881 10.1016/j.neuroimage.2011.10.018 PMC3254728

[68] Siegel JS, Power JD, Dubis JW, Vogel AC, Church JA, Schlaggar BL, Statistical improvements in functional magnetic resonance imaging analyses produced by censoring high-motion data points. Hum Brain Mapp. 2014;35(5):1981–96. 23861343 10.1002/hbm.22307 PMC3895106

[69] Power JD, Mitra A, Laumann TO, Snyder AZ, Schlaggar BL, Petersen SE. Methods to detect, characterize, and remove motion artifact in resting state fMRI. Neuroimage. 2014;84:320–41. 23994314 10.1016/j.neuroimage.2013.08.048 PMC3849338

[70] Power JD, Schlaggar BL, Petersen SE. Recent progress and outstanding issues in motion correction in resting state fMRI. Neuroimage. 2015;105:536–51. 25462692 10.1016/j.neuroimage.2014.10.044 PMC4262543

[71] Greene DJ, Church JA, Dosenbach NU, Nielsen AN, Adeyemo B, Nardos B, Multivariate pattern classification of pediatric Tourette syndrome using functional connectivity MRI. Dev Sci. 2016;19(4):581–98. 26834084 10.1111/desc.12407 PMC4945470

[72] Hoeft F, McCandliss BD, Black JM, Gantman A, Zakerani N, Hulme C, Neural systems predicting long-term outcome in dyslexia. Proc Natl Acad Sci U S A. 2011;108(1):361–6. 21173250 10.1073/pnas.1008950108 PMC3017159

[73] Yang H, Liu J, Sui J, Pearlson G, Calhoun VD. A Hybrid Machine Learning Method for Fusing fMRI and Genetic Data: Combining both Improves Classification of Schizophrenia. Front Hum Neurosci. 2010;4:192. 21119772 10.3389/fnhum.2010.00192 PMC2990459

[74] Bohlhalter S, Goldfine A, Matteson S, Garraux G, Hanakawa T, Kansaku K, Neural correlates of tic generation in Tourette syndrome: an event-related functional MRI study. Brain. 2006;129(Pt 8):2029–37. 16520330 10.1093/brain/awl050

[75] Hampson M, Tokoglu F, King RA, Constable RT, Leckman JF. Brain areas coactivating with motor cortex during chronic motor tics and intentional movements. Biol Psychiatry. 2009;65(7):594–9. 19111281 10.1016/j.biopsych.2008.11.012 PMC2679868

[76] Barnes KA, Cohen AL, Power JD, Nelson SM, Dosenbach YB, Miezin FM, Identifying basal ganglia divisions in individuals using resting-state functional connectivity MRI. Front Syst Neurosci. 2010;4:18. 20589235 10.3389/fnsys.2010.00018 PMC2892946

[77] Greene DJ, Laumann TO, Dubis JW, Ihnen SK, Neta M, Power JD, Developmental changes in the organization of functional connections between the basal ganglia and cerebral cortex. J Neurosci. 2014;34(17):5842–54. 24760844 10.1523/JNEUROSCI.3069-13.2014 PMC3996213

[78] Biederman J, Petty CR, Day H, Goldin RL, Spencer T, Faraone SV, Severity of the aggression/anxiety-depression/attention child behavior checklist profile discriminates between different levels of deficits in emotional regulation in youth with attention-deficit hyperactivity disorder. J Dev Behav Pediatr. 2012;33(3):236–43. 22278125 10.1097/DBP.0b013e3182475267 PMC3319866

[79] Althoff RR, Verhulst FC, Rettew DC, Hudziak JJ, van der Ende J. Adult outcomes of childhood dysregulation: a 14-year follow-up study. J Am Acad Child Adolesc Psychiatry. 2010;49(11):1105–16. 20970698 10.1016/j.jaac.2010.08.006 PMC2965164

[80] Eisenberg N, Spinrad TL. Emotion-related regulation: sharpening the definition. Child Dev. 2004;75(2):334–9. 15056187 10.1111/j.1467-8624.2004.00674.x

[81] Dosenbach NU, Nardos B, Cohen AL, Fair DA, Power JD, Church JA, Prediction of individual brain maturity using fMRI. Science. 2010;329(5997):1358–61. 20829489 10.1126/science.1194144 PMC3135376

[82] Greene DJ, Roller JM, Robichaux-Viehoever A, Bihun EC, Schlaggar BL, Black KJ. Reward enhances tic suppression in children within months of tic disorder onset. Dev Cogn Neurosci. 2015;11:65–74. 25220075 10.1016/j.dcn.2014.08.005 PMC4323948

[83] Martino D, Leckman JF. Tourette Syndrome. New York: Oxford University Press; 2013.

[84] Leckman JF, Cohen DJ. Tourette’s syndrome -- tics, obsessions, compulsions: Developmental psychopathology and clinical care. New York (US): John Wiley & Sons, Inc.; 1999.

[85] Freyer T, Valerius G, Kuelz AK, Speck O, Glauche V, Hull M, Test-retest reliability of event-related functional MRI in a probabilistic reversal learning task. Psychiatry Res. 2009;174(l):40–6. 19783412 10.1016/j.pscychresns.2009.03.003

[86] Constantino JN, Davis SA, Todd RD, Schindler MR, Gross MM, Brophy SL, Validation of a brief quantitative measure of autistic traits: Comparison of the Social Responsiveness Scale with the Autism Diagnostic Interview-Revised. J Autism Dev Disord. 2003;33(4):427–33. 12959421 10.1023/a:1025014929212

[87] Varni JW, Seid M, Rode CA. The PedsQL: measurement model for the pediatric quality of life inventory. Med Care. 1999;37(2):126–39. 10024117 10.1097/00005650-199902000-00003

[88] Leckman JF, Riddle MA, Hardin MT, Ort SI, Swartz RL, Stevenson J, The Yale Global Tic Severity Scale: initial testing of a clinician-rated scale of tic severity. J Am Acad Child Adolesc Psychiatry. 1989;28(4):566–73. 2768151 10.1097/00004583-198907000-00015

[89] Scahill L, Riddle MA, McSwiggin-Hardin M, Ort SI, Ring RA, Goodman WR, Children’s Yale-Brown Obsessive Compulsive Scale: reliability and validity. J Am Acad Child Adolesc Psychiatry. 1997;36(6):844–52. 9183141 10.1097/00004583-199706000-00023

[90] Conners CR, Sitarenios G, Parker JD, Epstein JN. The revised Conners’ Parent Rating Scale (CPRS-R): factor structure, reliability, and criterion validity. J Abnorm Child Psychol. 1998;26(4):257–68. 9700518 10.1023/a:1022602400621

[91] Robertson MM, Banerjee S, Rurlan R, Cohen DJ, Leckman JF, McMahon W, The Tourette syndrome diagnostic confidence index: development and clinical associations. Neurology. 1999;53(9):2108–12. 10599790 10.1212/wnl.53.9.2108

[92] Woods DW, Piacentini J, Himle MB, Chang S. Premonitory Urge for Tics Scale (PUTS): initial psychometric results and examination of the premonitory urge phenomenon in youths with tic disorders. J Dev Behav Pediatr. 2005;26(6):397–403. 16344654 10.1097/00004703-200512000-00001

[93] Goetz CG, Tanner CM, Wilson RS, Shannon RM. A rating scale for Gilles de la Tourette’s syndrome: description, reliability, and validity data. Neurology. 1987;37(9):1542–4. 3476860 10.1212/wnl.37.9.1542

[94] Goetz CG, Pappert EJ, Louis ED, Raman R, Leurgans S. Advantages of a modified scoring method for the Rush Video-Based Tic Rating Scale. Mov Disord. 1999;14(3):502–6. 10348478 10.1002/1531-8257(199905)14:3<502::aid-mds1020>3.0.co;2-g

[95] Goetz CG, Leurgans S, Chmura TA. Home alone: Methods to maximize tic expression for objective videotape assessments in Gilles de la Tourette syndrome. Mov Disord. 2001;16(4):693–7. 11481693 10.1002/mds.1159

[96] Barnea-Goraly N, Weinzimer SA, Ruedy KJ, Mauras N, Beck RW, Marzelli MJ, High success rates of sedation-free brain MRI scanning in young children using simple subject preparation protocols with and without a commercial mock scanner--the Diabetes Research in Children Network (DirecNet) experience. Pediatr Radiol. 2014;44(2):181–6. 24096802 10.1007/s00247-013-2798-7 PMC3946760

[97] Dosenbach NUF, Kolier JM, Earl EA, Miranda-Dominguez O, Klein RL, Van AN, Real-time motion analytics during brain MRI improve data quality and reduce costs. Neuroimage. 2017;161:80–93. 28803940 10.1016/j.neuroimage.2017.08.025 PMC5731481

[98] Tisdall MD, Reuter M, Qureshi A, Buckner RL, Fischl B, van der Kouwe AJW. Prospective motion correction with volumetric navigators (vNavs) reduces the bias and variance in brain morphometry induced by subject motion. Neuroimage. 2016;127:11–22. 26654788 10.1016/j.neuroimage.2015.11.054 PMC4754677

[99] Wang J, Licht DJ, Jahng GH, Liu CS, Rubin JT, Haselgrove J, Pediatric perfusion imaging using pulsed arterial spin labeling. J Magn Reson Imaging. 2003;18(4):404–13. 14508776 10.1002/jmri.10372

[100] Wang L, Lee DY, Bailey E, Hartlein JM, Gado MH, Miller MI, Validity of large-deformation high dimensional brain mapping of the basal ganglia in adults with Tourette syndrome. Psychiatry Res Neuroimaging. 2007;154(2):181–90. 10.1016/j.pscychresns.2006.08.006 PMC2859464 17289354

[101] Perantie DC, Wu J, Koller JM, Lim A, Warren SL, Black KJ, Regional brain volume differences associated with hyperglycemia and severe hypoglycemia in youth with type 1 diabetes. Diabetes Care. 2007;30(9):2331–7. 17575089 10.2337/dc07-0351

[102] Hershey T, Perantie DC, Wu J, Weaver PM, Black KJ, White NH. Hippocampal volumes in youth with type 1 diabetes. Diabetes. 2010;59(1):236–41. 19833895 10.2337/db09-1117 PMC2797927

[103] Perantie DC, Koller JM, Weaver PM, Lugar HM, Black KJ, White NH, Prospectively determined impact of type 1 diabetes on brain volume during development. Diabetes. 2011;60(11):3006–14. 21953611 10.2337/db11-0589 PMC3198062

[104] Fox MD, Snyder AZ, Vincent JL, Raichle ME. Intrinsic fluctuations within cortical systems account for intertrial variability in human behavior. Neuron. 2007;56:171–84. 17920023 10.1016/j.neuron.2007.08.023

[105] Fox MD, Snyder AZ, Vincent JL, Corbetta M, Van Essen DC, Raichle ME. The human brain is intrinsically organized into dynamic, anticorrelated functional networks. Proc Natl Acad Sci U S A. 2005;102(27):9673–8. 15976020 10.1073/pnas.0504136102 PMC1157105

[106] Fair DA, Dosenbach NU, Church JA, Cohen AL, Brahmbhatt S, Miezin FM, Development of distinct control networks through segregation and integration. Proc Natl Acad Sci U S A. 2007;104(33):13507–12. 17679691 10.1073/pnas.0705843104 PMC1940033

[107] Van Dijk KR, Sabuncu MR, Buckner RL. The influence of head motion on intrinsic functional connectivity MRI. Neuroimage. 2012;59(1):431–8. 21810475 10.1016/j.neuroimage.2011.07.044 PMC3683830

[108] Dosenbach NU, Fair DA, Miezin FM, Cohen AL, Wenger KK, Dosenbach RA, Distinct brain networks for adaptive and stable task control in humans. Proc Natl Acad Sci U S A. 2007;104(26):11073–8. 17576922 10.1073/pnas.0704320104 PMC1904171

[109] Field AP. Meta-analysis of correlation coefficients: a Monte Carlo comparison of fixed- and random-effects methods. Psychol Methods. 2001;6(2):161–80. 11411440 10.1037/1082-989x.6.2.161

[110] Salvador R, Suckling J, Schwarzbauer C, Bullmore E. Undirected graphs of frequency-dependent functional connectivity in whole brain networks. Philos Trans R Soc Lond B Biol Sci. 2005;360(1457):937–46. 16087438 10.1098/rstb.2005.1645 PMC1854928

[111] Karrer B, Levina E, Newman ME. Robustness of community structure in networks. Phys Rev E Stat Nonlin Soft Matter Phys. 2008;77(4 Pt 2):046119. 18517702 10.1103/PhysRevE.77.046119

[112] Honey C, Kotter R, Breakspear M, Sporns O. Network structure of cerebral cortex shapes functional connectivity on multiple time scales. Proc Natl Acad Sci U S A. 2007;104(24):10240–5. doi: 10.1073/pnas.0701519104 17548818 PMC1891224

[113] Fair DA, Cohen AL, Power JD, Dosenbach NU, Church JA, Miezin FM, Functional brain networks develop from a “local to distributed” organization. PLoS Comput Biol. 2009;5(5):e1000381. 19412534 10.1371/journal.pcbi.1000381 PMC2671306

[114] Bender-deMoll S, McFarland DA. The art and science of dynamic network visualization. J Soc Struct. 2006;7(2).

[115] Dosenbach NUF, Fair DA, Cohen AL, Schlaggar BL, Petersen SE. A dualnetworks architecture of top-down control. Trends Cogn Sci. 2008;12(3):99–105. 18262825 10.1016/j.tics.2008.01.001 PMC3632449

[116] Nielsen AN, Barch DM, Petersen SE, Schlaggar BL, Greene DJ. Machine learning with neuroimaging: Evaluating its applications in psychiatry. Biol Psychiatry Cogn Neurosci Neuroimaging. 2019;S2451-9022(19)30304-0. doi: 10.1016/j.bpsc.2019.11.007 PMC8746222 31982357

[117] Nielsen AJ, Gratton C, Church JA, Dosenbach NUF, Black KJ, Petersen SE, Atypical functional connectivity In Tourette Syndrome differs between children and adults. Biol Psychiatry. 2020;87(2):164–73. 31472979 10.1016/j.biopsych.2019.06.021 PMC6925331

[118] Nielsen AN, Kim S, Gratton C, Church JA, Black KJ, Petersen SE, Age-dependent differences in functional brain networks are atypical in Tourette syndrome. MedRxiv:2020:2020.04.06.20049817 [Preprint] 2020 4 07 Available from: https://www.medrxiv.org/content/10.1101/2020.04.06.20049817v1.full.pdf+html. Accessed 2020 May 21.

[119] Schölkopf B,J Smola A. Learning with kernels: support vector machines, regularization, optimization and beyond. Cambridge, Massachusetts (US): MIT Press; 2002 1 1 p. 626.

[120] Jäkel F, Scholkopf B, Wichmann F. Does Cognitive Science Need Kernels? Trends Cogn Sci. 2009;13(9):381–8. 19729333 10.1016/j.tics.2009.06.002

[121] Ben-Hur A, Ong CS, Sonnenburg S, Scholkopf B, Ratsch G. Support vector machines and kernels for computational biology. PLoS Comput Biol. 2008;4(10):e1000173. 18974822 10.1371/journal.pcbi.1000173 PMC2547983

[122] Naumovich Vapnik V Statistical learning theory. New York (US): John Wiley & Sons; 1998 1 1 p. 736.

[123] Weston J, Elisseefi A, Bakir GH, Sinz FH. The Spider machine learning toolbox 2005 Available from: https://web.archive.org/web/20200413222733/https://people.kyb.tuebingen.mpg.de/spider/mainframe.html. Accessed 2020 May 27.

[124] Kim S, Greene DJ, Bihun EC, Roller JM, Hampton JM, Acevedo H, Provisional Tic Disorder is not so transient. Sci Rep. 2019;9(1):3951. 30850688 10.1038/s41598-019-40133-4 PMC6408476

[125] Black KJ. DSM-5 misses an edge case in tic disorders nosology: Case report. F1000Res. 2020 Unpubbshed work. 10.12688/f1000research.23991.1 PMC7309051 32595960

[126] Yerys BE, Jankowski KF, Shook D, Rosenberger LR, Barnes KA, Berl MM, The fMRI success rate of children and adolescents: typical development, epilepsy, attention deficit/hyperactivity disorder, and autism spectrum disorders. Hum Brain Mapp. 2009;30(10):3426–35. 19384887 10.1002/hbm.20767 PMC2748172

[127] Conelea CA, Wellen B, Woods DW, Greene DJ, Black KJ, Specht M, Patterns and Predictors of Tic Suppressibility in Youth With Tic Disorders. Front Psychiatry. 2018;9:188. 29875706 10.3389/fpsyt.2018.00188 PMC5974106

[128] Kim S, Greene DJ, Robichaux-Viehoever A, Bihun EC, Roller JM, Acevedo H, Tic suppression in children with recent-onset tics predicts 1-year tic outcome. J Child Neurol. 2019;34(12):757–64. 31241402 10.1177/0883073819855531 PMC6733613

[129] Kim S, Greene DJ, Reiersen AM, Robichaux-Viehoever A, Bihun EC, Roller JM, Exploring Behavioral Markers Predicting One-Year Tic Outcome in Children with Recent-Onset Tics. J Am Acad Child Adolesc Psychiatry. 2019;58(10):S188–S.

[130] Kim S, Greene DJ, Badke D’Andrea C, Bihun EC, Roller JM, O’Reilly B, Hippocampal volume in Provisional Tic Disorder predicts tic severity at 12-month follow-up. bioRxiv2020:2020.02.05.935908 [Preprint], 2020 2 7 Available from: https://www.biorxiv.org/content/10.1101/2020.02.05.935908v1.full Accessed 2020 May 21. 10.3390/jcm9061715 PMC7355974 32503289

[131] Peterson BS, Choi HA, Hao X, Amat JA, Zhu H, Whiteman R, Morphologic features of the amygdala and hippocampus in children and adults with Tourette syndrome. Arch Gen Psychiatry. 2007;64(11):1281–91. 17984397 10.1001/archpsyc.64.11.1281 PMC2291296

[132] Schapiro AC, Reid AG, Morgan A, Manoach DS, Verfaellie M, Stickgold R. The hippocampus is necessary for the consolidation of a task that does not require the hippocampus for initial learning. Hippocampus. 2019;29(11):1091–100. 31157946 10.1002/hipo.23101 PMC6791729

[133] Kim S, Jackson SR, Groom M, Jackson GM. Visuomotor learning and unlearning in children and adolescents with tourette syndrome. Cortex. 2018;109:50–9. 30292925 10.1016/j.cortex.2018.08.007

[134] Black JK, Roller JM, Black KJ. TicTimer software for measuring tic suppression. F1000Res. 2017;6:1560. 29375812 10.12688/f1000research.12327.1 PMC5770993

[135] Vachon MJ, Striley CW, Gordon MR, Schroeder ML, Bihun EC, Roller JM, V1SIT-TS: A multimedia tool for population studies on tic disorders. F1000Res. 2016;5:1518. 27853509 10.12688/f1000research.7196.1 PMC5089139

[136] Csernansky JG, Wang L, Jones D, Rastogi-Cruz D, Posener JA, Heydebrand G, Hippocampal deformities in schizophrenia characterized by high dimensional brain mapping. Am J Psychiatry. 2002;159(12):2000–6. 12450948 10.1176/appi.ajp.159.12.2000

[137] Khan AR, Wang L, Beg MF. FreeSurfer-initiated fully-automated subcortical brain segmentation in MRI using Large Deformation Diffeomorphic Metric Mapping. Neuroimage. 2008;41(3):735–46. 18455931 10.1016/j.neuroimage.2008.03.024 PMC2905149

[138] Bennett SM, Capriotti M, Bauer C, Chang S, Keller AE, Walkup J, Development and open trial of a psychosocial intervention for young children with chronic tics: The CBIT-JR Study. Behav Ther. 10.1016/j.beth.2019.10.004 32586437

[139] Verdellen CW, Keijsers GP, Cath DC, Hoogduin CA. Exposure with response prevention versus habit reversal in Tourettes’s syndrome: a controlled study. Behav Res Ther. 2004;42(5):501–11. 15033497 10.1016/S0005-7967(03)00154-2

[140] Black JK, Black KJ. Software for web-based tic suppression training. F1000Res. 2017;6:2150. 30228859 10.12688/f1000research.13460.1 PMC6117856

